# Does pay-for-performance design matter? Evidence from Brazil

**DOI:** 10.1093/heapol/czae025

**Published:** 2024-04-25

**Authors:** Letícia Xander Russo, Timothy Powell-Jackson, Josephine Borghi, Juliana Sampaio, Garibaldi Dantas Gurgel Junior, Helena Eri Shimizu, Adriana Falangola Benjamin Bezerra, Keila Silene de Brito E Silva, Jorge Otávio Maia Barreto, André Luis Bonifácio de Carvalho, Roxanne J Kovacs, Luciano Bezerra Gomes, Nasser Fardousi, Everton Nunes da Silva

**Affiliations:** Faculty of Business, Accounting and Economics, Federal University of Grande Dourados, Rodovia Dourados—Itahum, Km 12, Dourados, MS 79804-970, Brazil; Department of Global Health and Development, London School of Hygiene and Tropical Medicine, London WC1E 7HT, United Kingdom; Department of Global Health and Development, London School of Hygiene and Tropical Medicine, London WC1E 7HT, United Kingdom; Department of Health Promotion, Federal University of Paraiba, João Pessoa 58051-900, Brazil; Oswaldo Cruz Foundation, Recife 50740-465, Brazil; Department of Collective Health, University of Brasilia, Brasilia 70910-900, Brazil; Department of Social Medicine, Federal University of Pernambuco, Recife 50670-901, Brazil; Collective Health Nucleous, Academic Center of Vitória, Federal University of Pernambuco, Vitória de Santo Antão 55608-680, Brazil; Oswaldo Cruz Foundation, Brasília 70904-130, Brazil; Department of Health Promotion, Federal University of Paraiba, João Pessoa 58051-900, Brazil; Department of Global Health and Development, London School of Hygiene and Tropical Medicine, London WC1E 7HT, United Kingdom; Department of Health Promotion, Federal University of Paraiba, João Pessoa 58051-900, Brazil; Department of Global Health and Development, London School of Hygiene and Tropical Medicine, London WC1E 7HT, United Kingdom; Faculty of Ceilândia, University of Brasilia, Brasilia 72220-275, Brazil

**Keywords:** Pay-for-performance, design features, incentive typology, primary care, Brazil, PMAQ

## Abstract

Pay-for-performance (P4P) schemes have been shown to have mixed effects on health care outcomes. A challenge in interpreting this evidence is that P4P is often considered a homogenous intervention, when in practice schemes vary widely in their design. Our study contributes to this literature by providing a detailed depiction of incentive design across municipalities within a national P4P scheme in Brazil [Primary Care Access and Quality (PMAQ)] and exploring the association of alternative design typologies with the performance of primary health care providers. We carried out a nation-wide survey of municipal health managers to characterize the scheme design, based on the size of the bonus, the providers incentivized and the frequency of payment. Using OLS regressions and controlling for municipality characteristics, we examined whether each design feature was associated with better family health team (FHT) performance. To capture potential interactions between design features, we used cluster analysis to group municipalities into five design typologies and then examined associations with quality of care. A majority of the municipalities included in our study used some of the PMAQ funds to provide bonuses to FHT workers, while the remaining municipalities spent the funds in the traditional way using input-based budgets. Frequent bonus payments (monthly) and higher size bonus allocations (share of 20–80%) were strongly associated with better team performance, while who within a team was eligible to receive bonuses did not in isolation appear to influence performance. The cluster analysis showed what combinations of design features were associated with better performance. The PMAQ score in the ‘large bonus/many workers/high-frequency’ cluster was 8.44 points higher than the ‘no bonus’ cluster, equivalent to a difference of 21.7% in the mean PMAQ score. Evidence from our study shows how design features can potentially influence health provider performance, informing the design of more effective P4P schemes.

Key messages

- The high heterogeneity in scheme design appears to be related to the differences observed in the effects of P4P on health care outcomes.
- Our study shows that P4P design feature is highly associated with team performance. Frequent bonus payments and higher size bonus allocations were identified as the main drivers of performance.

## Introduction

Pay-for-performance (P4P) schemes have been shown to have mixed or inconclusive effects on health care utilization and the quality of care in low- and middle-income countries (LMICs) (Eijkenaar *et al*., 2013; Barreto, 2015; Wiysonge *et al*., 2017; Diaconu *et al*., 2021), with much of the evidence from primary health care (PHC) settings. One challenge in interpreting this evidence is that P4P schemes are often considered homogenous interventions, when in practice they vary substantively in their design along key dimensions that may matter for their impact (Gergen *et al*., 2017; Paul *et al*., 2021; Singh *et al*., 2021; Diaconu *et al*., 2022). Differences in design and implementation strategies plausibly reflect contextual differences across LMICs regarding health service delivery, workforce, health financing and administrative capacity (Bitton *et al*., 2019). Indeed, there is a growing literature that has sought to identify a typology for P4P designs (Conrad and Perry, 2009; Van Herck *et al*., 2010; Ogundeji *et al*., 2018). A recent review proposed a typology of P4P designs and identified substantial variation in scheme design across settings (Kovacs *et al*., 2020).

Key features of the design of P4P schemes include choices about what is incentivized (utilization, quality and cost), who is incentivized (individual, team), payment attributes (frequency, size, time-lag and use of money), the basis for payment (absolute, relative, tournament) and gaming safeguards (performance audit and penalties) (Kovacs *et al*., 2020). These design choices are expected to have implications for how health workers respond to P4P and to play out differently depending on the underlying structure of the health system. For example, larger financial incentives are anticipated to increase workers’ effort to reach the programme’s goals (Fardousi *et al*., 2022); bonus payments to teams may be more appropriate in cases of high interdependence among workers for care delivery (Eijkenaar, 2013). P4P design can be tailored to accommodate the right incentives to overcome the problems arising from asymmetric information between the principal (donor, government) and agents (managers and workers) (Varian, 2014; Singh *et al*., 2021).

The empirical literature identifies certain programme design features that may be associated with greater P4P effectiveness, such as payments for reaching a certain level of coverage and including adjustments for service quality and equity (Diaconu *et al*., 2021). Schemes incentivizing a wide range of indicators and stakeholders, with efficient verification systems, have been found to be less prone to unintended negative effects (Singh *et al*., 2021; Diaconu *et al*., 2022). However, the reporting of scheme design within the literature tends to be incomplete (Kovacs *et al*., 2020). Furthermore, most P4P schemes are homogenous within a particular programme setting, making it difficult to unpack the contribution of design features to health provider performance.

The National Programme for Improving Primary Care Access and Quality (PMAQ) was implemented in Brazil during a 9-year period, reaching around 95% of Brazilian municipalities (5323 municipalities) and FHTs (40 684 teams) by the third round of the programme (Oct. 2015 to Dec. 2019) (Russo *et al*., 2021). Under the PMAQ scheme, the Ministry of Health provided funds based on performance achieved by FHTs. However, due to the degree of decentralization within the health system, funds were transferred to municipalities rather than directly to FHTs. Municipalities had flexibility in how they could use the funds, generating variation in programme design across municipalities.

The PMAQ scheme adopted by Brazil provides an opportunity to investigate whether P4P design matters for improving performance in PHC. On the one hand, all municipalities were exposed to the same programme rules at the national level, in terms of what was incentivized, instruments for performance measurement, and volume of funding based on FHT performance (Kovacs *et al*., 2021). On the other hand, municipalities had autonomy to decide whether to use the funds to give financial bonuses to FHT workers, which health workers within teams to reward, and the frequency of payments. Those municipalities that opted not to disburse any financial bonuses to workers could invest in improving health facility infrastructure, drug supply and health worker training. Municipalities could also opt to disburse bonuses to FHTs and invest in health facilities. An added advantage of the setting is that Brazil has a rich set of publicly available databases providing municipal-level data on socioeconomic, demographic and political variables.

Despite growing research on PMAQ, evidence on the programme design at the municipal level and its effects is scarce, a knowledge gap that was recently emphasized in a systematic review (Resende *et al*., 2021). The existing literature has tended to focus on a single region in Brazil (Rodrigues *et al*., 2021), with the exception of one national study that assessed the effect of sharing bonuses with FHT on quality of care using national programme data (Fardousi *et al*., 2022).

Our study contributes to this literature by providing a more detailed depiction of incentive design across municipalities within PMAQ in Brazil and exploring the effect of alternative design typologies on performance in terms of the quality of PHC. This fine-grained analysis of scheme design relied on primary data we collected through a municipality survey. Evidence from our study can shed light on how design features affect performance, informing the design of more effective P4P schemes.

## Methods

### Study setting

PMAQ was implemented in the context of the Unified Health System (SUS), which has provided universal and comprehensive health care that is free at the point of service use since 1988 (Paim, 2018). SUS is funded by the three federative authorities (national, state and municipal governments) (Cerda *et al*., 2022). The Family Health Strategy (FHS) is the main model for primary health care within the SUS, including individual, family and community interventions based on the following attributes: (1) first-contact care; (2) continuity of care; (3) comprehensive care and (4) coordinated care when users need speciality services (Oliveira and Pereira, 2013). The FHS is implemented by the FHTs, including physicians, nurses, nurse assistants and community health agents. FHTs work in collaboration with Oral Health Teams (OHTs) and the Family Health Support Units (NASFs). OHTs include dentists and dentist assistants. NASFs can include additional workers not included in the FHTs and OHTs, such as psychologists, occupational therapists, physiotherapists, speech therapists, nutritionists, physical educator, paediatricians, gynaecologists, pharmacists and public health and social workers. All teams are supported by cleaners, administrative assistants, managers and security staff.

### The PMAQ programme

The rapid expansion of FHTs, covering more than half of the Brazilian population in the early 2010s, gave rise to concerns about the large variation in clinical practice (Brazil, 2015), including in access to medical tests and diagnosis procedures (test schedule and delay in receiving test results), drug availably at the health facility, gatekeeping in authorizing access to speciality care (Souza *et al*., 2008) and quality of services delivered for antenatal care (Anversa *et al*., 2012). A range of factors were found to drive this variation, including persistent socioeconomic inequalities across municipalities, available health facility infrastructure, health care worker turnover and extent of work organization and a lack of systematic performance evaluation processes (Medeiros *et al*., 2010; Cavalcanti *et al*., 2015). To address these issues, municipalities requested more funding for PHC from the Ministry of Health, which resulted in the emergence of PMAQ, involving the Ministry of Health disbursing additional funds to municipalities conditional on improvement in access and quality of PHC. PMAQ also introduced a set of comparable and transparent performance assessment instruments related to quality of care and access, taking into account the diversity of local settings across the country. On this basis, PMAQ sought to change the culture around evaluation and accountability, by involving and incentivizing health managers at all levels (federal, state and municipal), FHTs and users (Brazil, 2015).

Under the PMAQ scheme, performance was measured by means of three instruments (self-evaluation, monitoring indicators and external evaluation). Self-evaluation was conducted by the FHTs, measuring the perception of workers about health facility infrastructure, work organization and outcomes. Monitoring indicators reflected strategic areas defined by the Ministry of Health in collaboration with state and municipal health managers, including priority groups and conditions such as maternal and child care, chronic diseases (diabetes and hypertension), infection diseases (tuberculosis and leprosy) and mental health. External evaluation comprised several face-to-face interviews conducted *in loco* (every FHT enrolled in PMAQ), with primary healthcare managers, workers from the FHTs and service users. The questionnaires included hundreds of items, representing structure, process and outcome indicators. Those interviews were carried out by independent parties (universities). In the third PMAQ round, the external evaluation comprised 60% of the final performance score of the FHTs, followed by monitoring indicators (30%) and self-evaluation (10%). The evaluation process took place at the beginning of each PMAQ round, approximately every 2 years: round 1 was from November/2011 to March/2013, round 2 from April/2013 to September/2015 and round 3 from October/2015 to December/2019 (Kovacs *et al*., 2021).

Although the method of performance assessment and level of financial rewards were determined at the national level (same rules for all FHTs), PMAQ allowed a high level of flexibility in terms of local implementation (Fardousi *et al*., 2022). The Ministry of Health transferred PMAQ funds to municipalities since legal regulation did not allow it to disburse bonuses directly to FHTs. Municipalities then decided whether and how to allocate funds to FHT, and how much funds to allocate.

### Study design

The study design was a cross-sectional analysis of the association between P4P design features and quality of care performance. We used primary data from a nation-wide survey of municipal health managers to characterize the local scheme design. We combined these data on scheme design with secondary sources of data measuring FHT performance and municipality characteristics. We examined the extent to which each design feature was associated with FHT performance. Finally, we used cluster analysis to group municipalities into design typologies to capture potential interactions between design features related to performance.

### Data collection

To measure variation in the design of PMAQ at the municipal level, we conducted an online survey of all 5570 municipalities across the country directed at the head of health at the municipal level (*secretário de saúde*). The electronic mailing-list was provided by the National Council for Municipal Secretary in Health (CONASEMS) with whom we collaborated. For those municipalities that did not reply to our invitation to participate in the survey, we sent a reminder every 15 days between October and December 2019. Survey respondents were asked to characterize the scheme design according to three key design features: the size of the incentive as a share of PMAQ resources, the FHT workers incentivized and the frequency of payments made to them. The design features drew from a previously published P4P design typology (Ogundeji *et al*., 2018), and the assessment of the respondents was based on the situation during the third round of PMAQ (2016–2019).

### Variables

From the survey, we generated four variables on scheme design: whether the municipality used PMAQ funds to give any bonuses to FHT workers; the size of the bonus as a share of PMAQ resources (1–20%; 21–40%; 41–60%; 61–80% and 81–100%); the providers incentivized (1 = incomplete members of FHT; 2 = all members of FHT; and 3 = all members of FHT and other workers (NASF and staff)); and the frequency of payments (1 = no fixed schedule; 2 = low, rewards paid every year; 3 = middle, paid every 2 to 6 months; and 4 = high, paid every month).

To measure performance, we used two measures. The first approach used the absolute PMAQ scores from participating FHTs. This score ranges from 0 to 100. The PMAQ score reflects hundreds of structure, process and outcome indicators obtained from three types of evaluation: (1) external evaluation (60% of the final PMAQ score); (2) monitoring indicators (30%); and (3) self-evaluation (10%) (Kovacs *et al*., 2021). The Ministry of Health provided the PMAQ score. The second measure of performance was a binary variable, which took a value of 1 if team performance was classified as better (PMAQ score between 71 and 79) or best (PMAQ score between 80 and 100), and otherwise 0.

We considered a number of municipal characteristics as potential confounders, including: (1) the human development index (HDI) for municipalities, calculated by the Atlas of Human Development in Brazil (Brazil, 2023c); (2) average PMAQ funds received per FHT/month in round 1, a measure to control for potential influence of additional funds awarded at the start of PMAQ (Brazil, 2023b); population size of the municipality, categorized into three groups (≤50 000 inhabitants; >50 000 and ≤100 000 inhabitants; >100 000 inhabitants) (Brazil, 2023a); (4) urban population, defined as the proportion of the total population living in urban areas (Brazil, 2023a); (5) party coalition, a dummy indicating whether the mayor and the president had the same party affiliations (TSE, 2023) and (6) rounds, a categorical variable measuring if the municipality participated in the previous PMAQ rounds (1 = did not participate in any previous PMAQ round; 2 = participated only in one previous PMAQ round (round 1 or 2); 3 = participated in the two previous PMAQ rounds (round 1 and 2). A complete description of the variables can be found in the supplementary material (Table S1).

### Statistical analyses

We followed two approaches to examining the association between PMAQ design features and performance. The analysis in both approaches was done at the FHT level. First, using OLS, we regressed the PMAQ score on the design features while controlling for municipality covariates. We examined each design feature separately, before running a regression with the inclusion of all the design features. We ran equivalent regressions with our second performance measure, the binary measure of better/best performance, as the dependent variable. We adjusted the standard errors for clustering at the municipality level given that variation in scheme design was at this level. A key drawback with this approach is that it assumes design features influence performance in an additive way—that is, they act individually. It does not account for possible interactions or synergies between them. This provided the motivation for an alternative approach to analysis.

Second, we used cluster analysis to group municipalities according to design typologies (combinations of design features that were observed across municipalities). We employed the k-means method using different numbers of k clusters. The optimal number of clusters was determined by the Calinski and Harabasz pseudo-F index, where larger values of the index correspond to more distinct grouping (Milligan and Cooper, 1985). These results suggested there were four distinct P4P design typologies in which the municipalities could be grouped. This is in addition to the group of municipalities that gave no bonuses to FHT workers, which acted as the reference category. We then ran OLS and logit regressions to investigate the association between these design typologies and quality of care, controlling for municipality covariates and clustering the standard errors at the municipality level.

### Sensitivity analyses

We conducted a number of sensitivity analyses. First, we investigated whether design features and clusters were associated with poor performance. Poor performance was defined as a binary variable of teams classified as worst (score between 0 and 40) and worse (score between 41 and 60). Second, we re-estimated the association between PMAQ design and the FHT performance score at the municipality level. This was conducted for each of the design indicators separately and the design typology clusters. Finally, we also ran regressions for poor performance at the municipal level (the proportion of teams classified as worst and worse within a municipality).

## Results

We obtained 818 survey responses (14.7% of all Brazilian municipalities), of which 682 (83.7%) participated in the third round of PMAQ. We dropped seven municipalities with missing data, resulting in a final sample of 811 municipalities (675 in the third round). Although there was a large proportion of non-responses, our sample appears to be similar to the national population of municipalities enrolled in PMAQ on various characteristics. There were no statistically significant differences in the percentage of party affiliation for the mayor and the president (41% vs 40%, respectively) and PMAQ funding received at the first round of the programme per FHT (R$1995.03 vs R$1904.70). There were statistically significant differences on other variables, but the magnitudes were small: municipalities in our sample had lower average FHT performance scores compared to the national average (51.11 vs 57.69), a higher proportion of teams classified as best and better (20% vs 16%) and a lower proportion classified as worst and worse (35% vs 55%), smaller average population size (proportion with < 50 000 inhabitants) (91% vs 88%), urban population (61% vs 64%) and HDI (0.64 vs 0.66) (Table 1).

**Table 1.** Descriptive statistics of the teams and municipalities included in our study (sample) compared to those not included (non-sample), third round, Brazil

	Sample			Non-sample			
Variable	Obs	Mean/Proportion	Std. Dev.	Obs	Mean/Proportion	Std. Dev.	*P*-value
PMAQ score	811	51.11	25.28	4516	57.69	11.87	<0.001
PMAQ classification (best and better)	811	0.20		4516	0.16		0.0012
PMAQ classification (worst and worsen)	811	0.35		4516	0.55		<0.001
HDI	811	0.64	0.07	4754	0.66	0.07	0.0031
Population size							
≤50 000	736	0.91		4169	0.88		
>50 000 and ≤100 000	46	0.06		305	0.06		0.0045
>100 000	29	0.04		280	0.06		
PMAQ funds per FHT	811	1995.03	2047.70	4754	1904.70	1991.69	0.0897
Urban population	811	0.61	0.21	4754	0.64	0.22	0.0034
Party affiliations	811	0.41	0.49	4610	0.40	0.49	0.5855
Previous Round							
No round	153						
Round 1 or 2	232						
Round 1 and 2	426						

Note: All variables were measured at the municipal level, except for the performance that was measured at FHT level.

**Figure 1.**
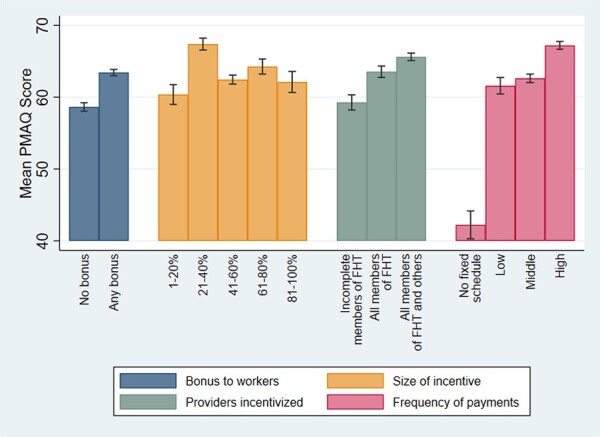
Average FHT performance by municipal design features (*n* = 5083), Brazil, third round of PMAQ

Note: FHT performance was based on the values calculated by the Ministry of Health, named PMAQ score.

Figure 1 describes the relationship between municipal incentive design features and FHT PMAQ scores. FHTs that received any share of the PMAQ bonus presented higher performance scores than those that received none, with teams in municipalities allocating 21–40% of total PMAQ resources obtaining the highest score. Teams in municipalities allocating bonus payments to all FHT members and other workers received higher scores than those allocating bonuses to only some team members. Teams in municipalities with a high frequency of payment (every month) also performed better than those with less frequent or unspecified payment frequencies.

Just under half of the study municipalities (302, 44.9%), containing 1849 FHTs, reported that they did not disburse bonuses to FHT members (cluster 1—no bonus). Remaining municipalities (373, 55.1%) were grouped into four clusters, representing different empirical combinations of size of incentive, providers incentivized and frequency of payments, according to the values of Calinski and Harabasz pseudo-F index (Table 2, Supplementary Material, Table S2).

- 45 municipalities provided a limited allocation of PMAQ resources (<40%) to a few workers (a subset of FHT members) with a high payment frequency (79% monthly) (cluster 2: small bonus/few workers/high frequency). This cluster is the closest to cluster 1 (no financial rewards to workers).
- 111 municipalities allocated a large share of PMAQ resources (mostly 40–100%) to a few workers (a subset of FHT members) with a relatively high frequency of payment (mostly from 1 to 6 months) (cluster 3: large bonus/few workers/high frequency).
- 105 municipalities provided a middle allocation of PMAQ resources (mostly 20–60%) to many workers (mostly all members of FH, OHT, NASF and/or staff) with a low frequency of payment (mostly from 2 to 12 months) (cluster 4: middle bonus/many workers/middle frequency).
- 122 municipalities provided a large allocation of PMAQ resources (mostly 40–80%) to many workers (mostly all members of FHT/OHT, NASF and/or staff) with a high frequency of payment (mostly monthly) (cluster 5: large bonus/many workers/high frequency).

**Table 2.** Variation in design features across the municipalities included in our sample and subgroups of design typology (clusters), Brazil, third round of PMAQ

	Design typology groups				
	Cluster 1 no bonus	Cluster 2 small bonus few workers high frequency	Cluster 3 large bonus few workers high frequency	Cluster 4 middle bonus many workers middle frequency	Cluster 5 large bonus many workers high frequency
	(*n* = 302), *n* (%)	(*n* = 45), *n* (%)	(*n* = 101), *n* (%)	(*n* = 105), *n* (%)	(*n* = 122), *n* (%)
Size of incentive in terms of PMAQ resources					
No bonus	302 (100)	0 (0)	0 (0)	0 (0)	0 (0)
1–20%	0 (0)	22 (49)	0 (0)	5 (5)	0 (0)
21–40%	0 (0)	23 (51)	0 (0)	19 (18)	12 (10)
41–60%	0 (0%)	0 (0)	52 (51)	78 (74)	89 (73)
61–80%	0 (0)	0 (0)	33 (33)	2 (2)	16 (13)
81–100%	0 (0)	0 (0)	16 (16)	1 (1)	5 (4)
Providers incentivized					
No bonus	302 (100)				
Incomplete members of FHT	0 (0)	33 (73)	77 (76)	3 (3)	0 (0)
All members of FHT	0 (0)	10 (22)	24 (24)	23 (22)	38 (31)
All members of FHT and others^a^	0 (0)	2 (4)	0 (0)	79 (75)	84 (69)
Frequency of payments					
No bonus	302 (100)				
No fixed schedule	0 (0)	3 (7)	2 (2)	7 (7)	0 (0)
Low (once per year)	0 (0)	6 (13)	6 (6)	36 (34)	0 (0)
Middle (every 2 to 6 months)	0 (0)	8 (18)	30 (30)	62 (59)	10 (8)
High (every month)	0 (0)	28 (62)	63 (62)	0 (0)	112 (92)

Note:

a Others can include NASF and/or staff.

Table 3 presents the regression results in which the design features enter additively. Any bonus payment was positively associated with performance relative to no bonus payment (columns 1 and 6). Teams allocating 21–40% and 61–80% as a bonus were also associated with a higher PMAQ score and a greater likelihood of being classified as best/better compared to those allocating 1–20% to bonuses in specifications that considered the size of bonus as the only design feature (models 2 and 7) and models including other design features (models 5 and 10). Bonus payment frequency was also a significant predictor of PMAQ score (models 4 and 5), but not of being classified as best/better. We found no association between the extent of providers incentivized and either outcome in any of the models. It is worth noting that the univariate relationships for each design feature (models 2–4; 7–9) are likely confounded by other design features. We also tested whether the coefficients of each indicator are simultaneously zero. It was found statistically different between all categories in the most complete specifications (F-test, models 5 and 10).

**Table 3.** Estimation results of the effect of individual design features on PMAQ performance score, analysis across family health teams (*n* = 5083), third round of PMAQ

	PMAQ score (OLS)					PMAQ classification (best/better) (logit)				
	(1)	(2)	(3)	(4)	(5)	(6)	(7)	(8)	(9)	(10)
Any bonus	4.791** (1.960)	0.625 (1.607)	2.495 (3.384)	−13.368*** (4.962)	−15.892*** (4.835)	0.681** (0.270)	−0.577 (0.361)	0.693** (0.342)	−0.781 (1.224)	−1.819 (1.204)
Size (ref = 1–20%)										
21–40%		7.572*** (1.722)			5.357*** (1.979)		1.828*** (0.362)			1.602*** (0.384)
41–60%		3.785* (2.000)			2.646 (1.862)		1.288*** (0.346)			1.100*** (0.342)
61–80%		8.509*** (2.313)			5.643*** (2.166)		1.669*** (0.406)			1.485*** (0.431)
81–100%		5.563** (2.610)			2.019 (2.234)		1.052*** (0.405)			0.751* (0.452)
Providers (ref = Incomplete members of FHT)										
All members of FHT			2.961 (3.461)		−0.822 (1.469)			0.024 (0.310)		−0.097 (0.284)
All members of FHT and others			4.876 (3.423)		2.684* (1.608)			0.208 (0.306)		0.371 (0.280)
Frequency (ref = No fixed schedule)										
Low				16.727*** (4.982)	14.392*** (4.965)				1.168 (1.245)	0.827 (1.269)
Middle				18.738*** (4.746)	16.272*** (4.897)				1.158 (1.195)	0.790 (1.258)
High				21.510*** (4.819)	19.653*** (4.855)				1.934 (1.194)	1.630 (1.229)
F-test (size)		5.62 (0.0002)			3.35 (0.0099)		29.67 (0.0000)			21.27 (0.0003)
F-test (providers)			1.96 (0.1421)		9.54 (0.0000)			0.69 (0.7088)		4.66 (0.0972)
F-test (frequency)				8.77 (0.0000)	4.64 (0.0099)				12.43 (0.0061)	14.38 (0.0024)
Observations	5083	5083	5083	5083	5083	5083	5083	5083	5083	5083
*R* ^2^	0.032	0.167	0.163	0.234	0.248	0.0164	0.068	0.0552	0.0753	0.0891

Notes: PMAQ score is defined as the score from the PMAQ participating teams for each municipality included in this study, ranging from 0 to 100. PMAQ classification is defined as a binary variable of family health teams classified as ‘best’ and ‘better’. Additional controls: HDI, population size, PMAQ funds per FHT, urban population, party affiliations and previous rounds of PMAQ.

Standard errors clustered at municipality level in parentheses.

*** *P* < 0.01,

** *P* < 0.05,

* *P* < 0.1.

Table 4 provides the results of regressions using design typologies derived from cluster analysis. Clusters 2, 3 and 5 are positively associated with better performance scores. Cluster 4 is not—and notably it is the only combination of design features without monthly payments, suggesting that monthly payments may matter for performance effects. Cluster 5 (large bonus/many workers/high-frequency) shows the strongest association with provider performance. It is worth noting, however, that the coefficients on clusters 2, 3 and 5 are somewhat similar, again suggesting that monthly payments may be a key driver of better performance. A broader conception of PHC teams (also including NASF and/or staff) seems to be the least relevant design feature, as cluster 4 is more likely to include all team members, yet there was no association between this design cluster and performance; it only becomes relevant when combined with high frequency payments or larger share allocations of PMAQ resources. These results were consistent across our two measures of primary healthcare performance (PMAQ score and the proportion of teams classified as best/better) (Table 4).

**Table 4.** Estimation of the effect of design clusters on performance across family health teams (*n* = 5083), third round of PMAQ

	PMAQ score (OLS)		PMAQ classification (logit)	
	(1)	(2)	(3)	(4)
PMAQ typology (ref = no bonus)				
2	7.046*** (2.358)	5.474*** (1.822)	0.992** (0.446)	0.774** (0.368)
3	5.284*** (1.724)	6.995*** (1.529)	0.645** (0.308)	0.876*** (0.293)
4	0.461 (3.926)	2.129 (2.566)	0.088 (0.374)	0.267 (0.380)
5	8.215*** (1.727)	8.437*** (1.555)	1.103*** (0.296)	1.216*** (0.293)
HDI		22.759*** (8.542)		4.363*** (1.534)
Population size		−1.277 (1.158)		−0.123 (0.188)
PMAQ funds per FHT		0.001*** (0.000)		0.000*** (0.000)
Urban population		−5.657* (3.127)		−1.060** (0.524)
Party Coalition		−0.909 (1.202)		−0.272 (0.197)
Rounds (PMAQ)		3.379** (1.481)		0.260** (0.131)
Constant	58.638*** (1.148)	38.825*** (5.135)	−1.553*** (0.229)	−4.453*** (0.916)
Observations	5083	5083	5083	5083
*R* ^2^	0.073	0.175	0.0368	0.0697

Standard errors clustered at municipality level in parentheses.

*** *P* < 0.01.,

** *P* < 0.05.,

* *P* < 0.1.

Overall, the results from the sensitivity analyses are in line with our main findings. Table S4 shows a negative association between the PMAQ design features and the likelihood of teams classified as worst/worse (the oppositive sign compared to the proportion of teams classified as best/better, as expected). The results using design typologies with negative performance outcomes are also similar (Table S5). When running analyses at the municipal level rather than the team level we also obtain similar findings (Tables S6–S9).

## Discussion

This study examined whether the PMAQ incentive design at the municipal level matters by analysing the association between key design features and team performance. We identified frequent, monthly, bonus payments and higher sized bonus allocations as the main drivers of team performance effects, which are supported by individual and cluster analyses (accounting for possible interactions between design features). Regarding the share of funds allocated as bonuses, our findings suggest a share of 20–80% was associated with higher performance, depending on the empirical strategy adopted. Increasing the number of providers incentivized beyond the FHT does not seem to affect performance.

A slim majority of the municipalities included in our study (373, 55.1%) opted for disbursing PMAQ resources to workers, using bonus allocations of different sizes, across different providers and with varying payment frequencies. The FHTs in those municipalities were generally associated with higher performance as measured by the PMAQ score compared to those in municipalities that did not disburse bonuses to workers (except cluster 4), with the most effective design cluster (5) also including regular performance payments. For example, the PMAQ score was 8.44 (95% CI 5.38–11.49, *P* < 0.001) higher in cluster 5 (Large-bonus/Many-workers/High-frequency) compared to cluster 1 (no bonus), which means an increase of 21.7% in the average PMAQ score (38.82). Therefore, our results suggest this P4P design feature is highly associated with performance outcomes.

Our findings about the effects of higher frequency payments on performance are supported by at least two economic principles (Eijkenaar *et al*., 2013). First, workers tend to discount future gains at an increasing rate over time, as the lag time between service delivery and payment increases reducing the present valuation of future payments. Second, there is a decreasing marginal utility rate for each additional unit of income for risk-averse workers. Based on that, a single large payment made at a given time will likely be less effective than a series of smaller, more frequent ones because each payment is recognized as a new reward rather than an addition to the previous gain.

It is well established that as the size of bonuses increases, ceteris paribus, workers tend to be more motivated to achieve performance targets. Our findings suggest that allocating some bonus to workers relative to no bonus is key, and the relative size of the bonus allocation has an effect, but this is not linear, with the 21–40% category having one of the largest effects on performance compared to municipalities allocating 1–20%. Further, there seems to be a threshold, beyond which additional bonuses may not substantially change behaviour (Ogundeji *et al*., 2018). Based on our study, this threshold seems to be over 80% of the total amount of PMAQ funding allocated to workers. In the Brazilian context, funding that was not allocated to workers was used to improve health facilities, and it seems that this allocation of funding was also important, particularly in deprived and remote areas. Appropriate health facility infrastructure may also contribute to wellbeing at work, which can affect workers’ productivity. However, new research is required to clarify the hypothesis we have raised.

The finding that transferring PMAQ resources as worker bonuses affects team performance supports the well-established notion that provider motivation is a key pathway through which P4P schemes will improve performance (Singh *et al*., 2021). However, in most municipalities 40–60% of PMAQ funds were invested in the health facility, improving infrastructure, accessibility, consultation privacy and availability of drugs and supplies. While our study cannot identify such benefits, it is likely these wider system investments contributed to increased performance, in settings like Brazil, with wide variation in facility capacity and service coverage, consistent with existing qualitative research (Lopes *et al*., 2015; Medrado *et al*., 2015; Feitosa *et al*., 2016; Bertusso and Rizzotto, 2018; Flôres *et al*., 2018; Singh *et al*., 2021).

Our study contributes to two previous studies of PMAQ design at the municipal level. The variation in scheme design was consistent with that identified through a survey conducted in Paraiba state (Rodrigues *et al*., 2021) across some domains: with 65.3% allocating 40–60% of the PMAQ resources to teams in Paraiba (compared to 58.7% in our study). There was also a broader perception of the team incentivized, including NASF and staff in both studies. However, only 15% of municipalities paid bonuses monthly compared to 54.4% in our study. There was no analysis seeking to investigate design features and performance in the Paraiba study. One previous study considered how scheme design in terms of the share of funds allocated as bonus affected team performance using routine data from 2346 municipalities (*modulo eletrônico*, in Portuguese), and a difference-in-differences approach (Fardousi *et al*., 2022), finding that teams from municipalities that disbursed PMAQ bonuses were associated with a 4.6 (95% CI: 2.7 to 6.4; *P* < 0.001) percentage point increase in the PMAQ score compared with non-bonus municipalities, and those with the largest bonus share had the greatest improvement [8.2 percentage points (95% CI: 6.2–10.2; *P* < 0.001)], which are within the range of estimates identified in our study (from 0 to 8.5 depending on the size of bonus disbursed). However, they were unable to consider the effect on performance of other design features, or identify the main design clusters.

Our study is limited in a number of respects. First, we used self-reported information about the PMAQ design collected via an electronic survey, which is susceptible to recall bias. Although we recognize that it might have some implications for the quality of our data, we believe it would be minimal. The third round of the programme was underway when we conducted our survey, thus responders answered questions about contemporaneous information at that time. Second, our sample represents about 15% of the Brazilian municipalities. Caution is needed when extrapolating the findings to other municipalities across the country, due to potential selection bias. However, our municipalities were broadly similar to the national average in terms of key characteristics. Third, although we use regression models to control for cross-sectional confounders, our findings rely on observational data, describing associations rather than causal relationships. Fourth, we used the PMAQ score and classification calculated by the Ministry of Health as a proxy for performance, which is mainly based on structural and process quality indicators. They might not reflect health outcomes among primary care users. Other studies have used hospitalizations for ambulatory care sensitive conditions (ACSCs) as a proxy for the effectiveness of the FHT, but they did not consider differences in PMAQ design (Araujo *et al*., 2017; Soares and Ramos, 2020; Fardousi *et al*., 2022). Finally, we were unable to determine how PMAQ funds that were not allocated as bonuses were used by municipalities and the extent and nature of investments in facilities, yet these investments may have been influential for performance.

Some implications for policy can be drawn from our study. First, P4P design features matter. Depending on the combination of design features, performance can increase or not. In our study, we estimated an increase of 21.7% in average performance from teams that received large bonus disbursed to many workers with high frequency of payment compared with teams that did not receive any bonus. Second, there are several ways to implement P4P scheme at the local level, even considering a few indicators as in our study. We identified five ways to implement PMAQ at the municipal level based on the combination of three indicators (size of incentive, workers incentivized and frequency of payment). Therefore, policy makers must be aware of potential heterogeneity at this stage.

PMAQ was replaced by a new P4P scheme in December 2019. Although the national-level design of the scheme has changed, with a reduced set of performance indicators (seven indicators) and more frequent four monthly performance measurement (Harzheim *et al*., 2020), municipalities still receive payments based on the performance of teams and have autonomy to determine whether and how much funds to disburse to workers. Future studies should continue to monitor the design choices made by municipalities and their effect on performance.

In conclusion, this study showed that P4P design features are highly associated with team performance. The combination of the design features is also associated with better/poor outcomes. We identified frequent bonus payments and higher size bonus allocations as the main drivers of performance. Our findings may contribute to more effective P4P schemes in LMICs.

## Supplementary Material

czae025_Supp

## Data Availability

The secondary data underlying this article were derived from sources in the public domain. The data sources are provided in the supplementary material. The survey data can be shared on reasonable request to the corresponding author.

## Reflexivity statement

This study involves international cooperation and researchers of multiple levels of seniority. The team includes seven females and seven males. The authors have different backgrounds and expertise (health economics, medicine, nursing, psychology and nutrition). Of them, 10 are residents of Brazil, including the principal investigator. All authors have in-depth knowledge of LMICs.
