# Supplementary material for: Does pay-for-performance design matter? Evidence from Brazil
Source: Health Policy Plan. 2024 Apr 25;39(6):593–602. doi: 10.1093/heapol/czae025 (PMC11145906; doi:10.1093/heapol/czae025)
Supplement: czae025_Supp [file czae025_supp.zip › Supplementary material.docx]

**Supplementary material**

**Table S1:** Description of variables used in the study.

| **Variable** | **Definition** | **Data availability** | **Source** |
| --- | --- | --- | --- |
| PMAQ clusters | P4P design implemented by the municipalities, based on three indicators: size of incentive, providers incentivized, and frequency of payments | Round 3 of PMAQ | Survey applied to Brazilian municipalities |
| PMAQ score | Average PMAQ score of PHC teams within a municipality | Round 3 of PMAQ | Primary Health Care Secretariat (SAPS), Ministry of Health |
| PMAQ classification (best and better) | Proportion of PMAQ teams classified as “best” and “better” | Round 3 of PMAQ | Primary Health Care Secretariat (SAPS), Ministry of Health |
| PMAQ classification (worst and worsen) | Proportion of PMAQ teams classified as “worst” and “worsen” | Round 3 of PMAQ | Primary Health Care Secretariat (SAPS), Ministry of Health |
| HDI | Human Development Index (HDI), composed of three indicators: life  expectancy, education, and income per capita. | 2010 | Atlas of Human Development in Brazil |
| Population size | Dummy broke down in three categories (=1 if ≤ 50,000 inhabitants;  =2 if >50,000 and ≤100,000 inhabitants; =3 if >100,000 inhabitants) | 2016 | Brazilian Institute of  Geography and Statistics (IBGE) |
| PMAQ funds per FHT | Average PMAQ funds received per FHT/month in round 1 | Round 1 of PMAQ | Survey |
| Urban population | Proportion of the total population living in urban areas | 2010 | Brazilian Institute of  Geography and Statistics (IBGE) |
| Party coalition | Dummy indicating if the party coalition from the mayor is the same of the President of Brazil (=1 if party coalition matches; =0 if otherwise). | 2016 | Superior Electoral Tribune (TSE) |
| Rounds | Previous experience in PMAQ rounds (1= if did not participate in any previous PMAQ round; =2 if participate only in one previous PMAQ round (round 1 or 2); =3 if participate in the two previous PMAQ rounds (round 1 and 2). | 2016 | Survey applied to Brazilian municipalities |

**Table S2:** Stopping-rule value for 3, 4, 5, 6 and 7 clusters

| Number of clusters | Calinski/Harabasz pseudo-F |
| --- | --- |
| 3 | 136.23 |
| **4** | **146.78** |
| 5 | 96.57 |
| 6 | 119.71 |
| 7 | 137.98 |

**Table S3:** Estimation results for PMAQ score using OLS and PMAQ classification (best and better) using logit, family health teams (n = 5,083), third round of PMAQ

|  |  |  | PMAQ Score | | | |  | PMAQ Classification | | | |
| --- | --- | --- | --- | --- | --- | --- | --- | --- | --- | --- | --- |
|  |  |  | (1) | (2) | (3) | (4) |  | (5) | (6) | (7) | (8) |
|  |  |  |  |  |  |  |  |  |  |  |  |
| Any bonus | |  | 0.625 | 2.495 | -13.368*** | -15.892*** |  | -0.577 | 0.693** | -0.781 | -1.819 |
|  |  |  | (1.607) | (3.384) | (4.962) | (4.835) |  | (0.361) | (0.342) | (1.224) | (1.204) |
| Size (ref=1-20%) | |  |  |  |  |  |  |  |  |  |  |
|  | 21-40% |  | 7.572*** |  |  | 5.357*** |  | 1.828*** |  |  | 1.602*** |
|  |  |  | (1.722) |  |  | (1.979) |  | (0.362) |  |  | (0.384) |
|  | 41-60% |  | 3.785* |  |  | 2.646 |  | 1.288*** |  |  | 1.100*** |
|  |  |  | (2.000) |  |  | (1.862) |  | (0.346) |  |  | (0.342) |
|  | 61-80% |  | 8.509*** |  |  | 5.643*** |  | 1.669*** |  |  | 1.485*** |
|  |  |  | (2.313) |  |  | (2.166) |  | (0.406) |  |  | (0.431) |
|  | 81-100% |  | 5.563** |  |  | 2.019 |  | 1.052*** |  |  | 0.751* |
|  |  |  | (2.610) |  |  | (2.234) |  | (0.405) |  |  | (0.452) |
| Providers (ref=Incomplete members of FHT) | |  |  |  |  |  |  |  |  |  |  |
|  | All members of FHT |  |  | 2.961 |  | -0.822 |  |  | 0.024 |  | -0.097 |
|  |  |  |  | (3.461) |  | (1.469) |  |  | (0.310) |  | (0.284) |
|  | All members of FHT and others |  |  | 4.876 |  | 2.684* |  |  | 0.208 |  | 0.371 |
|  |  |  |  | (3.423) |  | (1.608) |  |  | (0.306) |  | (0.280) |
| Frequency (ref=No fixed schedule) | |  |  |  |  |  |  |  |  |  |  |
|  | Low |  |  |  | 16.727*** | 14.392*** |  |  |  | 1.168 | 0.827 |
|  |  |  |  |  | (4.982) | (4.965) |  |  |  | (1.245) | (1.269) |
|  | Middle |  |  |  | 18.738*** | 16.272*** |  |  |  | 1.158 | 0.790 |
|  |  |  |  |  | (4.746) | (4.897) |  |  |  | (1.195) | (1.258) |
|  | High |  |  |  | 21.510*** | 19.653*** |  |  |  | 1.934 | 1.630 |
|  |  |  |  |  | (4.819) | (4.855) |  |  |  | (1.194) | (1.229) |
| HDI |  |  | 19.354*** | 21.250*** | 19.350** | 18.720** |  | 3.815*** | 4.168*** | 3.489** | 3.304** |
|  |  |  | (7.423) | (7.971) | (8.174) | (7.946) |  | (1.307) | (1.481) | (1.434) | (1.424) |
| Population size | |  | -1.562 | -1.396 | -0.513 | -0.763 |  | -0.187 | -0.141 | -0.070 | -0.133 |
|  |  |  | (1.216) | (1.210) | (1.002) | (1.017) |  | (0.202) | (0.196) | (0.192) | (0.197) |
| PMAQ funds per FHT | |  | 0.001*** | 0.001*** | 0.001*** | 0.001*** |  | 0.000*** | 0.000*** | 0.000*** | 0.000*** |
|  |  |  | (0.000) | (0.000) | (0.000) | (0.000) |  | (0.000) | (0.000) | (0.000) | (0.000) |
| Urban population | |  | -5.607* | -6.953** | -3.765 | -4.063 |  | -0.897* | -1.142** | -0.730 | -0.672 |
|  |  |  | (3.264) | (3.465) | (2.853) | (2.844) |  | (0.486) | (0.521) | (0.506) | (0.504) |
| Party affiliations | |  | -0.873 | -0.806 | -1.274 | -1.596 |  | -0.264 | -0.223 | -0.225 | -0.280 |
|  |  |  | (1.163) | (1.117) | (1.066) | (1.067) |  | (0.191) | (0.186) | (0.199) | (0.198) |
| Previous Round | |  | 3.813** | 3.137** | 1.613*** | 1.605*** |  | 0.297** | 0.270** | 0.133 | 0.132 |
|  |  |  | (1.745) | (1.289) | (0.610) | (0.580) |  | (0.147) | (0.138) | (0.119) | (0.115) |
| Constant | |  | 40.363*** | 41.533*** | 42.912*** | 44.142*** |  | -4.212*** | -4.286*** | -3.861*** | -3.645*** |
|  |  |  | (4.656) | (4.667) | (4.448) | (4.402) |  | (0.833) | (0.916) | (0.838) | (0.832) |
|  |  |  |  |  |  |  |  |  |  |  |  |
| F-test (size) | |  | 5.62 |  |  | 3.35 |  | 29.67 |  |  | 21.27 |
|  |  |  | (0.0002) |  |  | (0.0099) |  | (0.0000) |  |  | (0.0003) |
| F-test (providers) | |  |  | 1.96 |  | 9.54 |  |  | 0.69 |  | 4.66 |
|  |  |  |  | (0.1421) |  | (0.0000) |  |  | (0.7088) |  | (0.0972) |
| F-test (frequency) | |  |  |  | 8.77 | 4.64 |  |  |  | 12.43 | 14.38 |
|  |  |  |  |  | (0.0000) | (0.0099) |  |  |  | (0.0061) | (0.0024) |
|  |  |  |  |  |  |  |  |  |  |  |  |
| Observations | |  | 5,083 | 5,083 | 5,083 | 5,083 |  | 5,083 | 5,083 | 5,083 | 5,083 |
| R-squared | |  | 0.167 | 0.163 | 0.234 | 0.248 |  | 0.068 | 0.0552 | 0.0753 | 0.0891 |
| Standard errors clustered at municipality level in parentheses | | | |  |  |  |  |  |  |  |  |
| *** p<0.01, ** p<0.05, * p<0.1 | |  |  |  |  |  |  |  |  |  |  |

**Table S4:** Estimation results for PMAQ classification (worst and worsen) using logit, family health teams (n = 5,083), third round of PMAQ

|  |  | (1) | (2) | (3) | (4) |
| --- | --- | --- | --- | --- | --- |
|  |  |  |  |  |  |
| Any bonus | | 0.144 | -0.324 | 1.737* | 2.232** |
|  |  | (0.320) | (0.349) | (0.985) | (1.033) |
| Size (ref=1-20%) | |  |  |  |  |
|  | 21-40% | -1.482*** |  |  | -1.297*** |
|  |  | (0.403) |  |  | (0.425) |
|  | 41-60% | -0.665** |  |  | -0.470 |
|  |  | (0.339) |  |  | (0.382) |
|  | 61-80% | -1.199*** |  |  | -0.890** |
|  |  | (0.399) |  |  | (0.411) |
|  | 81-100% | -0.823* |  |  | -0.354 |
|  |  | (0.423) |  |  | (0.441) |
| Providers (ref=Incomplete members of FHT) | |  |  |  |  |
|  | All members of FHT |  | -0.251 |  | 0.145 |
|  |  |  | (0.392) |  | (0.249) |
|  | All members of FHT and others |  | -0.628* |  | -0.494* |
|  |  |  | (0.371) |  | (0.257) |
| Frequency (ref=No fixed schedule) | |  |  |  |  |
|  | Low |  |  | -2.005** | -1.507 |
|  |  |  |  | (1.009) | (0.987) |
|  | Middle |  |  | -2.380** | -1.977** |
|  |  |  |  | (0.979) | (0.971) |
|  | High |  |  | -2.784*** | -2.503** |
|  |  |  |  | (0.993) | (0.974) |
| HDI |  | -2.378* | -2.807* | -2.641* | -2.507* |
|  |  | (1.384) | (1.566) | (1.563) | (1.486) |
| Population size | | 0.114 | 0.095 | -0.008 | 0.040 |
|  |  | (0.156) | (0.164) | (0.153) | (0.154) |
| PMAQ funds per FHT | | -0.000*** | -0.000*** | -0.000*** | -0.000*** |
|  |  | (0.000) | (0.000) | (0.000) | (0.000) |
| Urban population | | 0.550 | 0.798 | 0.429 | 0.434 |
|  |  | (0.498) | (0.545) | (0.501) | (0.491) |
| Party affiliations | | 0.175 | 0.167 | 0.227 | 0.284 |
|  |  | (0.175) | (0.179) | (0.178) | (0.182) |
| Previous Round | | -0.410** | -0.324** | -0.170* | -0.157* |
|  |  | (0.186) | (0.143) | (0.092) | (0.090) |
| Constant | | 2.330*** | 2.242** | 2.189*** | 1.957** |
|  |  | (0.773) | (0.877) | (0.798) | (0.779) |
|  |  |  |  |  |  |
| F-test (size) | | 15.68 |  |  | 16.80 |
|  |  | (0.0035) |  |  | (0.0021) |
| F-test (providers) | |  | 4.00 |  | 7.29 |
|  |  |  | (0.1356) |  | (0.0262) |
| F-test (frequency) | |  |  | 13.50 | 18.49 |
|  |  |  |  | (0.0037) | (0.0003) |
|  |  |  |  |  |  |
| Observations | | 5,083 | 5,083 | 5,083 | 5,083 |
| R-squared | | 0.0677 | 0.0618 | 0.0881 | 0.1023 |
| Standard errors clustered at municipality level in parentheses | | | | | |
| *** p<0.01, ** p<0.05, * p<0.1 | |  |  |  |  |

**Table S5:** Estimation results for PMAQ classification (worst and worsen) using logit, family health teams (n = 5,083), third round of PMAQ

|  |  | (1) | (2) |
| --- | --- | --- | --- |
|  |  |  |  |
| PMAQ typology (ref=no bonus) | |  |  |
|  | 2 | -0.874* | -0.706** |
|  |  | (0.452) | (0.348) |
|  | 3 | -0.509*** | -0.754*** |
|  |  | (0.194) | (0.195) |
|  | 4 | -0.109 | -0.272 |
|  |  | (0.381) | (0.270) |
|  | 5 | -1.043*** | -1.133*** |
|  |  | (0.288) | (0.246) |
| HDI |  |  | -2.908* |
|  |  |  | (1.544) |
| Population size | |  | 0.080 |
|  |  |  | (0.162) |
| PMAQ funds per FHT | |  | -0.000*** |
|  |  |  | (0.000) |
| Urban population | |  | 0.632 |
|  |  |  | (0.523) |
| Party coalition | |  | 0.189 |
|  |  |  | (0.186) |
| Rounds (PMAQ) | |  | -0.352** |
|  |  |  | (0.161) |
| Constant | | -0.021 | 2.529*** |
|  |  | (0.123) | (0.834) |
|  |  |  |  |
| Observations | | 5,083 | 5,083 |
| R-squared | | 0.0296 | 0.067 |
| Standard errors clustered at municipality level in parentheses | | | |
| *** p<0.01, ** p<0.05, * p<0.1 | |  |  |

**Table S6:** Estimation results for PMAQ score and classification (best and better) using OLS, municipalities (n=675), third round of PMAQ

|  |  |  | Score | | | |  | Classification | | | |
| --- | --- | --- | --- | --- | --- | --- | --- | --- | --- | --- | --- |
|  |  |  | (1) | (2) | (3) | (4) |  | (5) | (6) | (7) | (8) |
|  |  |  |  |  |  |  |  |  |  |  |  |
| Any bônus | |  | 2.739* | 5.965*** | 1.561 | -1.337 |  | -0.010 | 0.157*** | -0.043 | -0.154* |
|  |  |  | (1.497) | (1.232) | (2.932) | (2.829) |  | (0.053) | (0.038) | (0.082) | (0.082) |
| Size (ref=1-20%) | |  |  |  |  |  |  |  |  |  |  |
|  | 21-40% |  | 5.894*** |  |  | 4.437** |  | 0.206*** |  |  | 0.167** |
|  |  |  | (1.762) |  |  | (1.845) |  | (0.067) |  |  | (0.066) |
|  | 41-60% |  | 5.673*** |  |  | 3.863** |  | 0.222*** |  |  | 0.183*** |
|  |  |  | (1.528) |  |  | (1.662) |  | (0.056) |  |  | (0.056) |
|  | 61-80% |  | 4.757** |  |  | 3.155 |  | 0.217*** |  |  | 0.188** |
|  |  |  | (2.129) |  |  | (2.185) |  | (0.072) |  |  | (0.073) |
|  | 81-100% |  | 2.678 |  |  | 1.162 |  | 0.109 |  |  | 0.060 |
|  |  |  | (2.327) |  |  | (2.400) |  | (0.083) |  |  | (0.087) |
| Providers (ref=Incomplete members of FHT) | |  |  |  |  |  |  |  |  |  |  |
|  | All members of FHT |  |  | 1.061 |  | 0.636 |  |  | -0.006 |  | -0.026 |
|  |  |  |  | (1.353) |  | (1.350) |  |  | (0.049) |  | (0.049) |
|  | All members of FHT and others |  |  | 3.141** |  | 3.167** |  |  | 0.061 |  | 0.062 |
|  |  |  |  | (1.228) |  | (1.252) |  |  | (0.043) |  | (0.045) |
| Frequency (ref=No fixed schedule) | |  |  |  |  |  |  |  |  |  |  |
|  | Low |  |  |  | 2.965 | 0.601 |  |  |  | 0.145 | 0.080 |
|  |  |  |  |  | (3.115) | (3.047) |  |  |  | (0.091) | (0.096) |
|  | Middle |  |  |  | 5.532* | 2.938 |  |  |  | 0.165* | 0.076 |
|  |  |  |  |  | (2.996) | (2.972) |  |  |  | (0.086) | (0.091) |
|  | High |  |  |  | 7.501** | 5.684* |  |  |  | 0.290*** | 0.227*** |
|  |  |  |  |  | (2.959) | (2.901) |  |  |  | (0.085) | (0.087) |
| HDI |  |  | 23.985*** | 21.311*** | 22.135*** | 21.635*** |  | 0.552** | 0.464** | 0.453** | 0.462** |
|  |  |  | (7.775) | (7.851) | (7.858) | (7.942) |  | (0.230) | (0.232) | (0.230) | (0.229) |
| Population size | |  | -0.518 | -0.508 | -0.209 | -0.502 |  | -0.008 | -0.004 | 0.005 | -0.006 |
|  |  |  | (0.866) | (0.839) | (0.826) | (0.833) |  | (0.027) | (0.027) | (0.028) | (0.027) |
| PMAQ funds per FHT | |  | 0.001*** | 0.001*** | 0.001*** | 0.001*** |  | 0.000*** | 0.000*** | 0.000*** | 0.000*** |
|  |  |  | (0.000) | (0.000) | (0.000) | (0.000) |  | (0.000) | (0.000) | (0.000) | (0.000) |
| Urban population | |  | -2.451 | -2.745 | -2.225 | -2.513 |  | -0.105 | -0.111 | -0.089 | -0.093 |
|  |  |  | (2.341) | (2.362) | (2.341) | (2.352) |  | (0.073) | (0.075) | (0.073) | (0.073) |
| Party affiliations | |  | -1.074 | -0.962 | -1.079 | -1.080 |  | -0.048* | -0.042 | -0.044* | -0.046* |
|  |  |  | (0.851) | (0.840) | (0.847) | (0.851) |  | (0.026) | (0.026) | (0.026) | (0.026) |
| Previous Round | |  | 1.344*** | 1.368*** | 1.304*** | 1.129** |  | 0.012 | 0.014 | 0.009 | 0.004 |
|  |  |  | (0.493) | (0.491) | (0.480) | (0.484) |  | (0.015) | (0.015) | (0.015) | (0.015) |
| Constant | |  | 38.654*** | 40.560*** | 39.461*** | 40.774*** |  | -0.203 | -0.151 | -0.156 | -0.133 |
|  |  |  | (4.554) | (4.636) | (4.609) | (4.688) |  | (0.128) | (0.129) | (0.129) | (0.127) |
|  |  |  |  |  |  |  |  |  |  |  |  |
| F-test (size) | |  | 4.03 |  |  | 1.91 |  | 4.4 |  |  | 3.21 |
|  |  |  | (0.0031) |  |  | (0.1062) |  | (0.0016) |  |  | (0.0125) |
| F-test (providers) | |  |  | 3.67 |  | 4.1 |  |  | 1.55 |  | 2.28 |
|  |  |  |  | (0.0259) |  | (0.0169) |  |  | (0.2122) |  | (0.1035) |
| F-test (frequency) | |  |  |  | 5.24 | 5.97 |  |  |  | 6.91 | 6.86 |
|  |  |  |  |  | (0.0014) | (0.0005) |  |  |  | (0.0001) | (0.0001) |
|  |  |  |  |  |  |  |  |  |  |  |  |
| Observations | |  | 675 | 675 | 675 | 675 |  | 675 | 675 | 675 | 675 |
| R-squared | |  | 0.209 | 0.206 | 0.214 | 0.232 |  | 0.120 | 0.106 | 0.130 | 0.152 |
| Standard errors clustered at municipality level in parentheses | | | |  |  |  |  |  |  |  |  |
| *** p<0.01, ** p<0.05, * p<0.1 | |  |  |  |  |  |  |  |  |  |  |

**Table S7:** Estimation results for PMAQ classification (worst and worsen) using OLS, municipalities (n=675), third round of PMAQ

|  |  | (1) | (2) | (3) | (4) |
| --- | --- | --- | --- | --- | --- |
|  |  |  |  |  |  |
| Any bonus | | 0.014 | -0.142*** | 0.110 | 0.240 |
|  |  | (0.078) | (0.045) | (0.130) | (0.150) |
| Size (ref=1-20%) | |  |  |  |  |
|  | 21-40% | -0.316*** |  |  | -0.249*** |
|  |  | (0.085) |  |  | (0.086) |
|  | 41-60% | -0.249*** |  |  | -0.173** |
|  |  | (0.079) |  |  | (0.082) |
|  | 61-80% | -0.185** |  |  | -0.122 |
|  |  | (0.094) |  |  | (0.095) |
|  | 81-100% | -0.135 |  |  | -0.088 |
|  |  | (0.112) |  |  | (0.113) |
| Providers (ref=Incomplete members of FHT) | |  |  |  |  |
|  | All members of FHT |  | -0.012 |  | 0.006 |
|  |  |  | (0.053) |  | (0.052) |
|  | All members of FHT and others |  | -0.138*** |  | -0.124*** |
|  |  |  | (0.045) |  | (0.045) |
| Frequency (ref=No fixed schedule) | |  |  |  |  |
|  | Low |  |  | -0.238* | -0.135 |
|  |  |  |  | (0.138) | (0.142) |
|  | Middle |  |  | -0.316** | -0.216 |
|  |  |  |  | (0.131) | (0.136) |
|  | High |  |  | -0.357*** | -0.286** |
|  |  |  |  | (0.131) | (0.134) |
| HDI |  | -0.402 | -0.281 | -0.341 | -0.327 |
|  |  | (0.285) | (0.289) | (0.283) | (0.286) |
| Population size | | -0.005 | -0.004 | -0.019 | -0.006 |
|  |  | (0.031) | (0.030) | (0.031) | (0.030) |
| PMAQ funds per FHT | | -0.000*** | -0.000*** | -0.000*** | -0.000*** |
|  |  | (0.000) | (0.000) | (0.000) | (0.000) |
| Urban population | | 0.038 | 0.049 | 0.032 | 0.048 |
|  |  | (0.092) | (0.092) | (0.092) | (0.092) |
| Party affiliations | | 0.033 | 0.030 | 0.035 | 0.033 |
|  |  | (0.032) | (0.032) | (0.032) | (0.032) |
| Previous Round | | -0.025 | -0.025 | -0.023 | -0.017 |
|  |  | (0.019) | (0.019) | (0.019) | (0.019) |
| Constant | | 0.892*** | 0.804*** | 0.868*** | 0.818*** |
|  |  | (0.155) | (0.159) | (0.154) | (0.156) |
|  |  |  |  |  |  |
| F-test (size) | | 4.12 |  |  | 2.72 |
|  |  | (0.0026) |  |  | (0.0290) |
| F-test (providers) | |  | 6.42 |  | 6.16 |
|  |  |  | (0.0017) |  | (0.0022) |
| F-test (frequency) | |  |  | 3.64 | 4.05 |
|  |  |  |  | (0.0127) | (0.0072) |
|  |  |  |  |  |  |
| Observations | | 675 | 675 | 675 | 675 |
| R-squared | | 0.125 | 0.119 | 0.121 | 0.150 |
| Standard errors clustered at municipality level in parentheses | | |  |  |  |
| *** p<0.01, ** p<0.05, * p<0.1 | |  |  |  |  |

**Table S8:** Estimation results for PMAQ score and classification (best and better) using OLS, municipalities (n=675), third round of PMAQ

|  |  |  | PMAQ Score | |  | PMAQ Classification | |
| --- | --- | --- | --- | --- | --- | --- | --- |
|  |  |  | (1) | (2) |  | (3) | (4) |
|  |  |  |  |  |  |  |  |
| PMAQ typology (ref=no bonus) | |  |  |  |  |  |  |
|  | 2 |  | 5.527*** | 5.817*** |  | 0.107** | 0.111** |
|  |  |  | -1.416 | -1.384 |  | -0.054 | -0.052 |
|  | 3 |  | 4.151*** | 5.708*** |  | 0.116*** | 0.146*** |
|  |  |  | -1.232 | -1.294 |  | -0.038 | -0.039 |
|  | 4 |  | 5.852*** | 6.495*** |  | 0.088*** | 0.104*** |
|  |  |  | -1.044 | -1.054 |  | -0.034 | -0.034 |
|  | 5 |  | 10.834*** | 10.848*** |  | 0.301*** | 0.306*** |
|  |  |  | -1.079 | -1.093 |  | -0.037 | -0.037 |
| HDI |  |  |  | 20.742*** |  |  | 0.441* |
|  |  |  |  | -7.935 |  |  | -0.231 |
| Population size | |  |  | -0.561 |  |  | -0.008 |
|  |  |  |  | -0.861 |  |  | -0.028 |
| PMAQ funds per FHT | |  |  | 0.001*** |  |  | 0.000*** |
|  |  |  |  | 0 |  |  | 0 |
| Urban population | |  |  | -2.215 |  |  | -0.094 |
|  |  |  |  | -2.33 |  |  | -0.073 |
| Party coalition | |  |  | -1.063 |  |  | -0.047* |
|  |  |  |  | -0.834 |  |  | -0.025 |
| Rounds (PMAQ) | |  |  | 1.285*** |  |  | 0.01 |
|  |  |  |  | -0.488 |  |  | -0.015 |
| Constant | |  | 57.714*** | 40.930*** |  | 0.149*** | -0.131 |
|  |  |  | -0.634 | -4.677 |  | -0.016 | -0.129 |
|  |  |  |  |  |  |  |  |
| Observations | |  | 675 | 675 |  | 675 | 675 |
| R-squared | |  | 0.136 | 0.221 |  | 0.107 | 0.14 |
| Standard errors clustered at municipality level in parentheses | | | | |  |  |  |
| *** p<0.01, ** p<0.05, * p<0.1 | |  |  |  |  |  |  |

**Table S9:** Estimation results for PMAQ classification (worst and worsen) using OLS, municipalities (n=675), third round of PMAQ

|  |  | (1) | (2) |
| --- | --- | --- | --- |
|  |  |  |  |
| PMAQ typology (ref=no bonus) | |  |  |
|  | 2 | -0.110* | -0.110* |
|  |  | -0.061 | -0.062 |
|  | 3 | -0.095** | -0.122*** |
|  |  | -0.045 | -0.047 |
|  | 4 | -0.176*** | -0.184*** |
|  |  | -0.041 | -0.043 |
|  | 5 | -0.337*** | -0.333*** |
|  |  | -0.036 | -0.036 |
| HDI |  |  | -0.271 |
|  |  |  | -0.288 |
| Population size | |  | -0.002 |
|  |  |  | -0.031 |
| PMAQ funds per FHT | |  | -0.000*** |
|  |  |  | 0 |
| Urban population | |  | 0.028 |
|  |  |  | -0.091 |
| Party coalition | |  | 0.035 |
|  |  |  | -0.032 |
| Rounds (PMAQ) | |  | -0.021 |
|  |  |  | -0.019 |
| Constant | | 0.532*** | 0.796*** |
|  |  | -0.023 | -0.158 |
|  |  |  |  |
| Observations | | 675 | 675 |
| R-squared | | 0.100 | 0.133 |
| Standard errors clustered at municipality level in parentheses | | | |
| *** p<0.01, ** p<0.05, * p<0.1 | |  |  |
